# The Microbiology of Non-*aeruginosa Pseudomonas* Isolated From Adults With Cystic Fibrosis: Criteria to Help Determine the Clinical Significance of Non-*aeruginosa Pseudomonas* in CF Lung Pathology

**DOI:** 10.3389/bjbs.2022.10468

**Published:** 2022-06-08

**Authors:** John E. Moore, John McCaughan, Jacqueline C. Rendall, Beverley C. Millar

**Affiliations:** ^1^ Laboratory for Disinfection and Pathogen Elimination Studies, Northern Ireland Public Health Laboratory, Belfast City Hospital, Belfast, United Kingdom; ^2^ School of Medicine, Dentistry and Biomedical Sciences, The Wellcome-Wolfson Institute for Experimental Medicine, Queen’s University, Belfast, United Kingdom; ^3^ Department of Medical Microbiology, The Royal Group of Hospitals, Belfast, United Kingdom; ^4^ Northern Ireland Regional Adult Cystic Fibrosis Centre, Belfast City Hospital, Belfast, United Kingdom

**Keywords:** cystic fibrosis, *Pseudomonas aeruginosa*, microbiology, *Pseudomonas fluorescens*, *Pseudomonas putida*, *Pseudomonas stutzeri*, *Pseudomonas fragi*, *Pseudomonas oleovorans*

## Abstract

**Introduction:** There is a paucity of reports on non-*aeruginosa Pseudomonas* (NAPs) in cystic fibrosis, hence this study wished 1). to examine the diversity/frequency of NAPs in an adult CF population, 2) to compare/contrast the microbiology and genomics of NAPs to *P. aeruginosa* and 3) to propose clinical and laboratory criteria to help determine their clinical significance in CF lung pathology.

**Materials and Methods:** Microbiological data was examined from 100 adult patients with cystic fibrosis from birth to present (31/12/2021), equating to 2455 patient years. 16S rDNA phylogenetic relatedness of NAPs was determined, as well as bioinformatical comparison of whole genomes of *P. aeruginosa* against *P. fluorescens*.

**Results:** Ten species were isolated from this patient cohort during this time period, with three species, i.e., *P. fluorescens, P. putida* and *P. stutzeri*, accounting for the majority (87.5%) of non-*aeruginosa* reports. This is the first report of the isolation of *P. fragi, P. nitroreducens, P. oryzihabitans* and *P. veronii* in patients with cystic fibrosis. The mean time to first detection of any non-*aeruginosa* species was 183 months (15.25 years) [median = 229 months (19.1 years)], with a range from 11 months to 338 months (28.2 years). Several of the NAPs were closely related to *P. aeruginosa*.

**Discussion:** NAPs were isolated infrequently and were transient colonisers of the CF airways, in those patients with CF in which they were isolated. A set of ten clinical and laboratory criteria are proposed to provide key indicators, as to the clinical importance of the non-*aeruginosa* species isolated.

## Introduction

Cystic fibrosis (CF) is an autosomal recessive disease of mainly Caucasian populations of European ancestry, which is exacerbated by a continuous cycle of respiratory inflammation and lung infection, which may become chronic, leading to increasing disease severity (1). For a seminal review of the pathophysiology of the disease, please see Shteinberg et al. (1). The production of thick viscous sputum as a result in the physiological problems of transporting chloride ions allows for the entrapment of environmental bacterial and fungal organisms, which may eventually lead to chronic colonisation and infection. Therefore, people with cystic fibrosis are vulnerable to acquiring new environmental organisms in the lower respiratory tract, as well as clinical isolates from other patients with cystic fibrosis, through cross infection.

Several bacterial genera and species are particularly associated with increased morbidity and mortality in patients with cystic fibrosis, as detailed in Supplementary Material S1. Of these, *Pseudomonas aeruginosa* is the most clinically important Gram negative pathogen observed in cystic fibrosis (2). *Pseudomonas aeruginosa* is a Gram-negative non-fermenting rod with a taxonomical lineage, as shown in Supplementary Material S2. The genus is currently composed of 251 species, with a validly published and correct name, but has 342 described species, including synonyms (3). When species are included which do not have a validly published and correct name, the number of child taxa is 446. Supplementary Material S3 lists in alphabetical order all the species names within the genus *Pseudomonas*, with a link to further taxonomical information for each of these species.

Whilst *Pseudomonas aeruginosa* is the species most frequently isolated from the sputum of CF patients, other species of *Pseudomonas* have been isolated from CF patients (4, 5). Currently, there is a paucity of reports on these non-*aeruginosa Pseudomonas* (NAPs), hence it was the aim of this study to 1). examine the diversity and frequency of NAPs in an adult CF population and 2) compare and contrast the microbiology and genomics of NAPs isolated from the CF lung in comparison to *P. aeruginosa* and 3) to propose clinical and laboratory criteria that will help determine the clinical significance of non-*aeruginosa Pseudomonas* in CF lung pathology.

## Materials and Methods

### Patient Demographics and Microbiology Analyses

One hundred adult (≥18 years old) CF patients from the Northern Ireland Regional Adult Cystic Fibrosis Centre, Belfast City Hospital, with a diagnosis of CF, were included in this retrospective analysis for the presence of non-*aeruginosa* *Pseudomonas* (NAP) in their sputum at any time, since birth until the present (31 December 2021). Isolation of these NAP organisms was performed in accordance with microbiological Standard Operating Procedures detailed in the UK NHS.

UK Standards for Microbiology Investigations: Investigation of bronchoalveolar lavage, sputum and associated specimens (available at https://assets.publishing.service.gov.uk/government/uploads/system/uploads/attachment_data/file/800451/B_57i3.5.pdf) in a UKAS accredited NHS clinical microbiology laboratory. Sex and CFTR mutation type were noted for those CF patients who cultured NAPs organisms in their sputum on at least one occasion, during their lifetime. Time (months) to first isolation of a NAP organism was noted.

### Molecular and Genomic Analyses of Non-*aeruginosa Pseudomonas* Isolated From Adult CF Patients

####  16S rDNA Phylogenetic Comparison

Complete 16S rDNA sequences of the 10 NAPs, as well as *P. aeruginosa* were retrieved from NCBI Nucleotide (https://www.ncbi.nlm.nih.gov/nucleotide/), employing the following GenBank Accession numbers: *P. fragi* NR_024946, *P. oryzihabitans* NR_025881, *P. aeruginosa* NR_026078, *P. veronii* NR_028706, *P. nitroreducens* NR_042435, *P. alcaligenes* NR_043419, *P. fluorescens* NR_043420, *P. mendocina* NR_043421, *P. putida* NR_043424, *P. stutzeri* NR_103934 and *P. oleovorans* NR_114478. Sequences were aligned employing the Neighbor-Joining Tree method, using the Align/Assemble and Tree tools in Geneious Prime 2022.0.2 software.

#### Comparison of Whole Genome Maps of *P. fluorescens*, *P. putida*, *P. stutzeri* and *P. aeruginosa*

Whole genomes maps of *P. fluorescens* (NC_012660)*, P. putida* (NZ_CP016634), *P. stutzeri* (AP0244722) *and P. aeruginosa* PAO1 (NC_002516) were retrieved from NCBI Genome (https://www.ncbi.nlm.nih.gov/genome/).

#### Whole Genome Comparison of *P. aeruginosa* and *P. fluorescens*

A whole genome sequence comparison was performed with *P. fluorescens* (NC_012660) *and P. aeruginosa* PAO1 (NC_002516), employing Geneious Prime 2022.0.2 software, employing the Mauve genome alignment tool. This program has been used previously on fairly closely related members of *Gammaproteobacteria* (6). As previously described by Dikow (7), traditional multiple sequence alignment cannot be used on complete genome sequences because significant rearrangement of genes or fragments has been shown to occur over evolutionary history. Mauve addresses this issue by finding locally collinear blocks (LCBs), or contiguous segments of sequence within which there has not been rearrangement, but within a longer sequence that may have been subject to rearrangement events. The default parameters in Mauve were used.

## Results

### Patient Demographics and Microbiology Analyses

One hundred adult (>18 years old) patients with cystic fibrosis were examined in this study, with a mean age of 24.6 years, median age 24 years, with an age range of 18–76 years. This patient cohort consisted of 50 females and 50 males. Microbiological data was examined from 100 patients from birth to present (31/12/2021), equating to 2455 patient years.

Ten non-*aeruginosa Pseudomonas* species were isolated, as detailed in Table 1. The frequency of the occurrence of these species is shown in Supplementary Material S4. Patient demographics, CFTR mutation type and time to first detection for all NAP species is shown in Table 1. In some patients, two NAPs were detected in an individual patient (Supplementary Material S5), but three or more NAPs were not observed in a single patient. There were five patients where a NAP was isolated (3 x *P. fluorescens* + 2 x *P. putida*), who never isolated *P. aeruginosa*. Of the 33 patients who isolated *P. fluorescens*, two of these organisms were isolated prior to the patient isolating *P. aeruginosa*. Likewise 1 *P. stutzeri* and three *P. putida* organisms were isolated prior to the patient isolating *P. aeruginosa*.

**TABLE 1:** Description of non-*aeruginosa Pseudomonas* species isolated from adult patients with cystic fibrosis (CF) and associated characteristics.

Organism	Occurrence (%) in adult CF patients^a^ (*n* = 100)	Patient sex	Mean time to first isolation (months)	Median time to first isolation (months)	Time to first isolation (range [months])	F508del/F508del	F508del/other	other/other
*Pseudomonas fluorescens*	33	60.6% female/39.4% male	195	198	23–338	45.5	48.5	6
*Pseudomonas putida*	18	33% female/67% male	184	220	11–285	50	22	28
*Pseudomonas stutzeri*	6	33% female/67% male	171	170	90–273	83	17	
*Pseudomonas alcaligenes*	1	Female		284		100		
*Pseudomonas fragi*	1	Female		207		100		
*Pseudomonas mendocina*	1	Female		195		100		
*Pseudomonas nitroreducens*	1	Female		300				100
*Pseudomonas oleovorans*	1	Male		269		100		
*Pseudomonas oryzihabitans*	1	Female		160			100	
*Pseudomonas veronii*	1	Female		286		100		
Mean			183	229				

a since birth–equivalent to 2455 patient years.

A microbiological comparison of the non-*aeruginosa* species isolated including description, morphology, optimum temperature, ability to reduce nitrates, habitat, pathological association with infection, % G + C and genome size (Mb) is shown (Supplementary Material S6).

### Molecular and Genomic Analyses of Non-*aeruginosa Pseudomonas* Isolated From Adult CF Patients

####  16S rDNA Phylogenetic Comparison

Phylogenetic relatedness between the 10 NAPs species and *P. aeruginosa* is shown in Supplementary Material S7. An alignment of rDNA 16S sequences from the 11 species (10 non-*aeruginosa* species + *P. aeruginosa*) was performed using the NCBI BLAST alignment tool (https://blast.ncbi.nlm.nih.gov/Blast.cgi) and the resulting consensus alignment has now been deposited in GenBank, with the Accession Number OM653502 (see Supplemetary Material S8), to support future bioinformatics investigation and primer design.

#### Comparison of Whole Genome Maps of *P. fluorescens*, *P. putida*, *P. stutzeri* and *P. aeruginosa*

Whole genomes maps of *P. fluorescens* (NC_012660)*, P. putida* (NZ_CP016634), *P. stutzeri* (AP0244722) *and P. aeruginosa* PAO1 (NC_002516) are shown in Supplementary Materials S9–S12, respectively.

#### Whole Genome Comparison of *P. aeruginosa* and *P. fluorescens*

**FIGURE 1:**
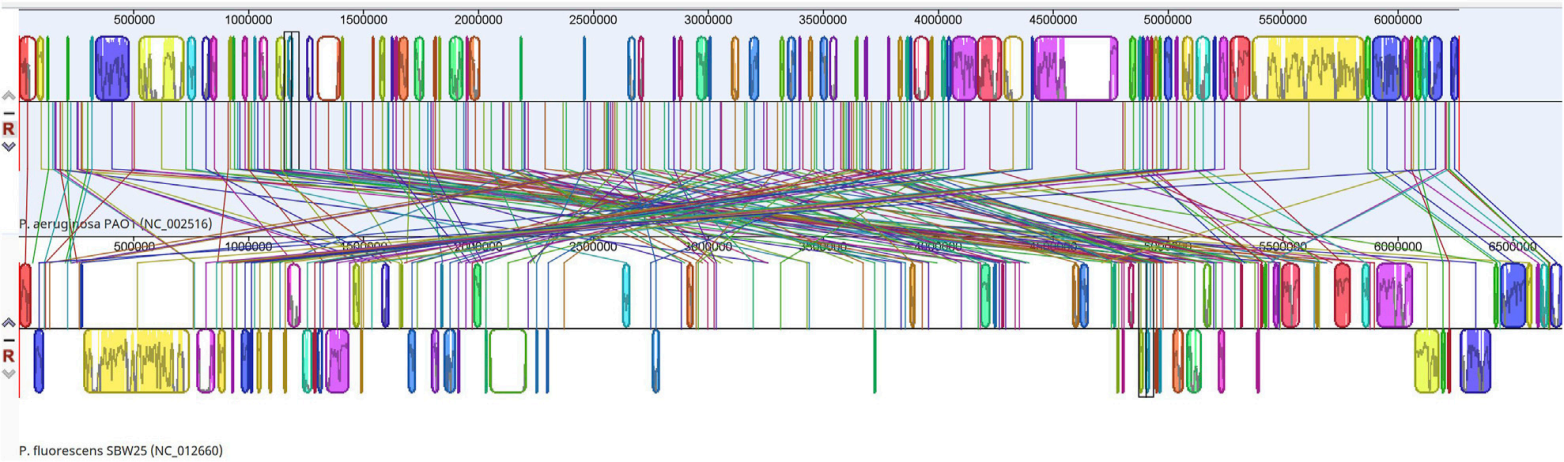
Whole genome alignment map of *P. aeruginosa* PAO1 (NC_002516) with *P. fluorescens* (NC_012660) revealing 196 locally collinear blocks conserved among these two species. Each chromosome has been laid out horizontally and homologous blocks in each genome are shown as identically coloured regions linked across genomes.

Putatively homologous regions were found across the two whole genomes examined, 6,264,404 bp (*P. aeruginosa*) and 6,722,539 bp (*P. fluorescens*). Each sequence is represented by one horizontal panel of blocks. Each coloured block represents a region of sequence that aligns to part of the other genome and is presumably homologous and free from internal rearrangement. 196 Locally Co-linear Blocks (LCBs) were generated between the two genomes (Figure 1) and a representative of the annotations in an LCB (LCB#27) is shown (Supplementary Material S13). Supplementary Material S14 displays annotations of coding DNA sequences (CDS) from Locally Collinear Block (LCB) #27 of a genome comparison between *Pseudomonas aeruginosa* PAO1 (NC_002516) and *Pseudomonas fluorescens* (SBW25 (NC_012660).

## Discussion

Cystic fibrosis lung pathology is dominated by the presence of the genus *Pseudomonas*. However, this dominance is due a single species, namely *Pseudomonas aeruginosa* and there is a paucity of information relating to the other non-*aeruginosa* species involvement in CF lung pathology or even their physical presence in the CF lung. Given that the genus is composed of at least 251 formally described and validated species (see Supplementary Materials S3), it was the aim of this study to examine the presence of other non-*aeruginosa* species within this genus that have also been reported in the sputum of adult patients with cystic fibrosis and to consider why other non-*aeruginosa* species do not play a clinically significant role in CF lung infection.

In this study, we examined the sputum microbiology of 100 adult CF patients from their birth until the present (31 December 2021), which in totality equated to 2455 patient years. Ten species were isolated from this patient cohort during this time period, with three species, i.e., *P. fluorescens, P. putida* and *P. stutzeri*, accounting for the majority (87.5%) of non-*aeruginosa* reports, with the remaining seven species being isolated sporadically from single patients. This is the first report of the isolation of *P. fragi, P. nitroreducens, P. oryzihabitans* and *P. veronii* in patients with cystic fibrosis. The mean time to first detection of any non-*aeruginosa* species was 183 months (15.25 years) [median = 229 months (19.1 years)], with a range from 11 months to 338 months (28.2 years). Supplementary Materials S15 shows the relationship between patient age and the first occurrence of any non-*aeruginosa* species. The patient age group with the most frequent occurrence of first isolate NAPs was the 11–16 age group. With regard to patient CFTR mutation type, nearly all (87%) had at least one F508del mutation (52% F508 del homozygous; 35% F508del heterozygous), with the remainder (13%) having other CFTR mutations.

The presence of *P. aeruginosa* in the sputum microbiology of any CF patient is of concern, given the correlation between the presence of this organism and clinical deterioration in the patient, with increased morbidity and mortality. However, should this clinical concern be extended to the other non-*aeruginosa* species, given that these species have a pedigree and legacy of belonging to the same genus, which is associated with such poor clinical outcomes? To address this, we firstly examined the published literature to determine if any of the non-*aeruginosa* species that we isolated had been described in CF previously (Table 2). Overall, these reports indicate the potential for laboratory misidentification but do not report these species to be a significant pathogen in CF, rather a transient coloniser of the CF lung.

**TABLE 2:** Previous reports on the involvement of non-*aeruginosa Pseudomonas* within cystic fibrosis (CF).

Organism	Description	References
*Pseudomonas fluorescens*	Isolation of *P. fluorescens* from respiratory tract cultures of cystic fibrosis (CF) patients	(8)
	Isolation of *P. fluorescens* from protected catheter brush and bronchoalveolar lavage specimens in a patient with bronchiectasis	(9)
	Development of a diagnostic PCR assay that targets a heat-shock protein gene (*groES*) for detection of *Pseudomonas* spp. in cystic fibrosis patients, including *P. fluorescens*	(10)
	In antibiotic susceptibility testing, the activity of ceftazidime was two dilutions greater than the other three cephalosporins against *P. fluorescens*	(11)
	Isolation of *P. fluorescens* from the sputum of seven different CF individuals from CF five different CF treatment centres across the United States (Hartford, CT; Seattle, WA; Salt Lake City, UT; Little Rock, AR; and Augusta, GA). Genomes were sequenced	(12, 13)
	Isolation of *P. fluorescens* from a 77 year old CF woman with F508del/3849 + 10kbC>T with moderate lung disease	
*Pseudomonas putida*	Recovered from oropharyngeal cultures from healthy, non-CF infants in 3 months–6 months age (1/21; 4.8%) and from 6 months to 9 months age group (1/20; 5%). Not isolated from oropharyngeal cultures from 75 CF infants in the first year of life. Not considered pathogenic. Care should be taken to not over interpret the presence of some of these organisms in the oropharyngeal cultures of asymptomatic CF infants	(14)
	Misidentification of *P. aeruginosa* by the MicroScan microbiology system. The most common misidentifications at 24 h were *P. fluorescens-P. putida* (i.e., the strain was either *P. fluorescens* or *P. putida*, but the system did not make the distinction and yielded the result of *P. fluorescens/putida* (n = 20). At 48 h, 12 of 20 (60%) *P.fluorescens-P. putida* misidentifications as *P. aeruginosa*	(15)
	Development of a diagnostic PCR assay that targets a heat-shock protein gene (*groES*) for detection of *Pseudomonas* spp. in cystic fibrosis patients, including *P. putida*	(10)
	Isolation of *P. fluorescens/P. putida* from respiratory tract cultures of cystic fibrosis (CF) patients. Colonization is considered sporadic	(8)
	Description of *P. putida’s* ability to form hyper-biofilm variants naturally. Whilst *P.putida* is not considered a pathogen in patients with CF, they could potentially aid and abeit the pathogenesis of *P. aeruginosa,* by offering protection to *P. aeruginosa* within their biofilm, thus challenging its role as a true commensal organism in CF.	(16)
*Pseudomonas stutzeri*	Development of a diagnostic PCR assay that targets a heat-shock protein gene (*groES*) for detection of *Pseudomonas* spp. in cystic fibrosis patients, including *P. putida*	(10)
	Presence of algT. Whilst *P. stutzeri* is not considered a pathogen in patients with CF, they could potentially aid and abeit the pathogenesis of *P. aeruginosa*, by offering protection to *P. aeruginosa* within their algT biofilm biochemistry	(17)
*Pseudomonas alcaligenes*	Misidentification of *P. alcaligenes* as *P. aeruginosa* employing molecular techniques. *P. alcaligenes* and *P. pseudoalcaligenes* are isolated infrequently from CF patients and are easily differentiated from *P. aeruginosa* by phenotypic tests	(18)
*Pseudomonas fragi*	No reports	
*Pseudomonas mendocina*	Misidentification of *P. aeruginosa* as *P. mendocina*	(19)
*Pseudomonas nitroreducens*	No reports	
*Pseudomonas oleovorans*	Case of metalworking fluids (MWFs)-Hypersensitivity pneumonitis sensitized to *Pseudomonas* oleovorans in a cystic fibrosis patient	(20)
*Pseudomonas oryzihabitans*	No reports	
*Pseudomonas veronii*	No reports	(5)
*Pseudomonas lundensis*	12 *P. lundensis* strains were isolated from the sputum of different cystic fibrosis patients	

The isolation of species of low pathogenesis and virulence from the genus *Pseudomonas* creates a dilemma for the clinical team when considering what clinical significance to place on the species and whether to treat with antibiotics or not. In order to help address such a dilemma, when have developed a set of ten clinical and laboratory criteria, as listed in Table 3. These criteria should be assessed qualitatively, both singly and collectively, in order to provide some key indicators to the clinical team, as to the clinical importance of the non-*aeruginosa* species isolated. The more attributes that align with these criteria, then the more likely that the isolated organism is clinically significant.

**TABLE 3:** Proposed criteria for evaluating the clinical significance of colonisation of non-*aeruginosa Pseudomonas* (NAP) organisms in CF lung pathology.

Criteria	Description
1	Persistent colonisation (intermittent & chronic) as shown by positive repeat sputum cultures for same organism, as defined by the Leeds criteria (21)
2	High quantitative counts in sputum (10^8^–10^9^ colony forming units/g sputum)
3	Reduction in lung function markers (FEV_1_ & FVC) in absence of known CF microbial pathogens or other clinical reason (e.g., poor adherence to airway clearance)
4	Worsening chest radiological imaging in absence of known CF microbial pathogens
5	Increase in C-reactive protein (CRP), without other focus of infection or in absence of known CF microbial pathogens
6	Clinical improvement and pro rata reduction in NAP organisms after commencement of antibiotic therapy
7	Ecological displacement of existing flora with NAP organisms
8	Serological response with specific antibody to NAP organisms as determined by counter immunoelectrophoresis (CIE)
9	Pathogenic pedigree: Has the NAP organism isolated been associated with NAP-related pathology previously and has known virulence determinants?
10	May evolve into phenotypes which are multi- and pan-resistant to antipseudomonal antibiotics, including aminoglycosides, beta-lactams and fluoroquinolones

FEV_1_ = forced expiratory volume in the first second.

FVC, forced vital capacity.

NAP, non-aeruginosa *Pseudomonas* species.

Within the *Gammaproteobacteria*, we observe that the genus *Pseudomonas* consists of several groups, including the *aeruginosa* group, the *chlororaphis* group, the *fluorescens* group, the *putida* group, the *stutzeri* group and the *syringae* group (available at http://lifemap-ncbi.univ-lyon1.fr/). The *aeruginosa* group is in turn composed of eight species, including *P. mendocina, P. alcaligenes, P. anguilliseptica, P. resinovorans, P. nitroreducens, P. furukawaii, P. oleovorans*, as well as *P. aeruginosa*. In our study, we were able to identify 4/8 non-*aeruginosa* species, which belonged to this group. Of the three remaining species (i.e., *P. anguilliseptica, P. resinovorans and P. furukawaii*)*,* none of these of these taxa have been reported in CF lung microbiology. This was confirmed in our study with close genetic distance as seen in the dendogram of relatedness, as seen in Supplementary Material S7.

### Study Limitations

This study involved examining sputum microbiology from birth to the present (December 2021), of a set of 100 adult CF patients from a single adult CF centre in the UK. Sputum microbiology methods followed UK Standard Operating Procedures in a UK accredited NHS Clinical Microbiology Laboratory which helped to standardise variability in methodology, to potentially account for the comparison of these data with data from other CF centres. Furthermore, the infection control practices at this single centre, as well as infection control-related health literacy of the CF patients may influence the cohort of NAPs isolated and reported in this study. Therefore, it would beneficial to expand this study in the future to include several other CF centres both locally, nationally and internationally.

So why do we not see the dominance of other species of *Pseudomonas*, other than *P. aeruginosa*, in patients with cystic fibrosis, even those that share the same taxonomic grouping as *P. aeruginosa*? This may be partly explained by the other non-*aeruginosa* species being less host-adapted than *P. aeruginosa*, to survival in a clinical host, which to many species, may be too alien a lifestyle compared to their natural free-living lifestyle in the environment. Factors including competition for nutrients, competition from a large microbiome microbial community within the lung, oxygen deprivation and survival in anaerobic microniches, as well as challenges from innate host defence mechanisms may select for the survival and eventual succession of *P. aeruginosa* as the ultimate climax ecological community. Secondly, NAPs may not be as ubiquitous in the environment as *P. aeruginosa*, so they may not have the same opportunities to come into physical interaction with this clinical environment to allow colonisation to occur. However this is unlikely given the extensive size of species members within the genus. Thirdly, NAPs may lack specific core oligosaccharide domains, which *P. aeruginosa* possess, which act as a ligand-receptor for binding to CFTR protein. Future studies should therefore aim to elucidate why non-*aeruginosa* species of *Pseudomonas* are poor bacterial pathogens in CF, which in turn could help our understanding as to why their close neighbour, *P. aeruginosa*, is such a successful pathogen.

## Summary Table

### What Is Known About This Topic

• *Pseudomonas aeruginosa* plays a significant role in lung pathology associated with people with cystic fibrosis (CF).
• There is a paucity of published information describing the microbiology of non-*aeruginosa* species of this genus in CF.

### What This Work Adds

• This study describes the diversity of non-*aeruginosa* species found in 100 people with CF, equating to 2455 patient years.
• A set of ten clinical and laboratory criteria are proposed to provide key indicators, as to the clinical importance of the non-*aeruginosa* species isolated.

## Data Availability

The datasets presented in this study can be found in online repositories. The names of the repository/repositories and accession number(s) can be found below: https://www.ncbi.nlm.nih.gov/genbank/, OM653502.

## References

[1] Shteinberg M Haq IJ Polineni D Davies JC. Cystic Fibrosis. Lancet (2021) 397(10290):2195–211. 10.1016/s0140-6736(20)32542-3 34090606

[2] Parkins MD Somayaji R Waters VJ. Epidemiology, Biology, and Impact of Clonal *Pseudomonas aeruginosa* Infections in Cystic Fibrosis. Clin Microbiol Rev (2018) 31(4):e00019–18. 10.1128/CMR.00019-18 30158299 PMC6148191

[3] Bacterio. LPSN - List of Prokaryotic Names with Standing in Nomenclature (2022). Available at: https://www.bacterio.net/ (Accessed February 17, 2022). 10.1093/nar/gkt1111 PMC3965054 24243842

[4] Fabbri A Tacchella A Manno G Viscoli C Palmero C Gargani GF. Emerging Microorganisms in Cystic Fibrosis. Chemioterapia (1987) 6(1):32–7. 3103930

[5] Scales BS Erb-Downward JR Falkowski NR LiPuma JJ Huffnagle GB. Genome Sequences of 12 *Pseudomonas Lundensis* Strains Isolated from the Lungs of Humans. Genome Announc (2018) 6(7):e01461–17. 10.1128/genomeA.01461-17 29449399 PMC5814480

[6] Darling AE Miklós I Ragan MA. Dynamics of Genome Rearrangement in Bacterial Populations. Plos Genet (2008) 4:e1000128–10. 10.1371/journal.pgen.1000128 18650965 PMC2483231

[7] Dikow RB. Genome-level Homology and Phylogeny of *Shewanella* (*Gammaproteobacteria: Lteromonadales: Shewanellaceae*). BMC Genomics (2011) 12:237. 10.1186/1471-2164-12-237 21569439 PMC3107185

[8] Klinger JD Thomassen MJ. Occurrence and Antimicrobial Susceptibility of Gram-Negative Nonfermentative Bacilli in Cystic Fibrosis Patients. Diagn Microbiol Infect Dis (1985) 3(2):149–58. 10.1016/0732-8893(85)90025-2 3979021

[9] Pang JA Cheng A Chan HS Poon D French G. The Bacteriology of Bronchiectasis in Hong Kong Investigated by Protected Catheter brush and Bronchoalveolar Lavage. Am Rev Respir Dis (1989) 139(1):14–7. 10.1164/ajrccm/139.1.14 2912333

[10] Clarke L Moore JE Millar BC Garske L Xu J Heuzenroeder MW Development of a Diagnostic PCR Assay that Targets a Heat-Shock Protein Gene (groES) for Detection of *Pseudomonas* Spp. In Cystic Fibrosis Patients. J Med Microbiol (2003) 52(9):759–63. 10.1099/jmm.0.05077-0 12909651

[11] Baltch AL Smith RP Ritz W. Comparative Antimicrobial Activity of FK037, Cefpirome, Ceftazidime and Cefepime against Aminoglycoside-Sensitive and Aminoglycoside-Resistant *Pseudomonas aeruginosa* and Pseudomonas Spp. Chemotherapy (1994) 40(6):391–8. 10.1159/000239298 7842822

[12] Scales BS Erb-Downward JR Huffnagle IM LiPuma JJ Huffnagle GB. Draft Genome Sequences of Seven *Pseudomonas Fluorescens* Subclade III Strains Isolated from Cystic Fibrosis Patients. Genome Announc (2015) 3(1):e01285–14. 10.1128/genomeA.01285-14 PMC4319496 25635025

[13] Heirali A McKeon S Purighalla S Storey DG Rossi L Costilhes G Assessment of the Microbial Constituents of the Home Environment of Individuals with Cystic Fibrosis (CF) and Their Association with Lower Airways Infections. PLoS One (2016) 11(2):e0148534. 10.1371/journal.pone.0148534 26859493 PMC4747485

[14] Carlson D McKeen E Mitchell M Torres B Parad R Comeau AM Oropharyngeal flora in Healthy Infants: Observations and Implications for Cystic Fibrosis Care. Pediatr Pulmonol (2009) 44(5):497–502. 10.1002/ppul.21029 19360845

[15] Saiman L Burns JL Larone D Chen Y Garber E Whittier S. Evaluation of MicroScan Autoscan for Identification of *Pseudomonas aeruginosa* Isolates from Cystic Fibrosis Patients. J Clin Microbiol (2003) 41(1):492–4. 10.1128/JCM.41.1.492-494.2003 12517904 PMC149561

[16] Xu A Zhang X Wang T Xin F Ma LZ Zhou J Rugose Small Colony Variant and its Hyper-Biofilm in *Pseudomonas aeruginosa*: Adaption, Evolution, and Biotechnological Potential. Biotechnol Adv (2021) 53:107862. 10.1016/j.biotechadv.2021.107862 34718136

[17] Goldberg JB Gorman WL Flynn JL Ohman DE. A Mutation in algN Permits Trans Activation of Alginate Production by algT in *Pseudomonas* Species. J Bacteriol (1993) 175(5):1303–8. 10.1128/jb.175.5.1303-1308.1993 8444793 PMC193215

[18] Ghozzi R Morand P Ferroni A Beretti J-L Bingen E Segonds C Capillary Electrophoresis-Single-Strand Conformation Polymorphism Analysis for Rapid Identification of *Pseudomonas aeruginosa* and Other Gram-Negative Nonfermenting Bacilli Recovered from Patients with Cystic Fibrosis. J Clin Microbiol (1999) 37(10):3374–9. 10.1128/JCM.37.10.3374-3379.1999 10488211 PMC85573

[19] Lagares A Agaras B Bettiol MP Gatti BM Valverde C. A Cultivation-independent PCR-RFLP Assay Targeting oprF Gene for Detection and Identification of *Pseudomonas* Spp. in Samples from Fibrocystic Pediatric Patients. J Microbiol Methods (2015) 114:66–74. 10.1016/j.mimet.2015.05.008 25960432

[20] Bellanger AP Morisse-Pradier H Reboux G Scherer E Pramil S Dominique S Hypersensitivity Pneumonitis in a Cystic Fibrosis Patient. Occup Med (Lond) (2019) 69(8-9):632–4. 10.1093/occmed/kqz115 31504833

[21] Lee TWR Brownlee KG Conway SP Denton M Littlewood JM. Evaluation of a New Definition for Chronic *Pseudomonas aeruginosa* Infection in Cystic Fibrosis Patients. J Cystic Fibrosis (2003) 2(1):29–34. 10.1016/S1569-1993(02)00141-8 15463843

