# Supplementary material for: The Microbiology of Non-aeruginosa Pseudomonas Isolated From Adults With Cystic Fibrosis: Criteria to Help Determine the Clinical Significance of Non-aeruginosa Pseudomonas in CF Lung Pathology
Source: Br J Biomed Sci. 2022 Jun 8;79:10468. doi: 10.3389/bjbs.2022.10468 (PMC9302546; doi:10.3389/bjbs.2022.10468)
Supplement: Supplementary file 1 [file datasheet7.pdf]

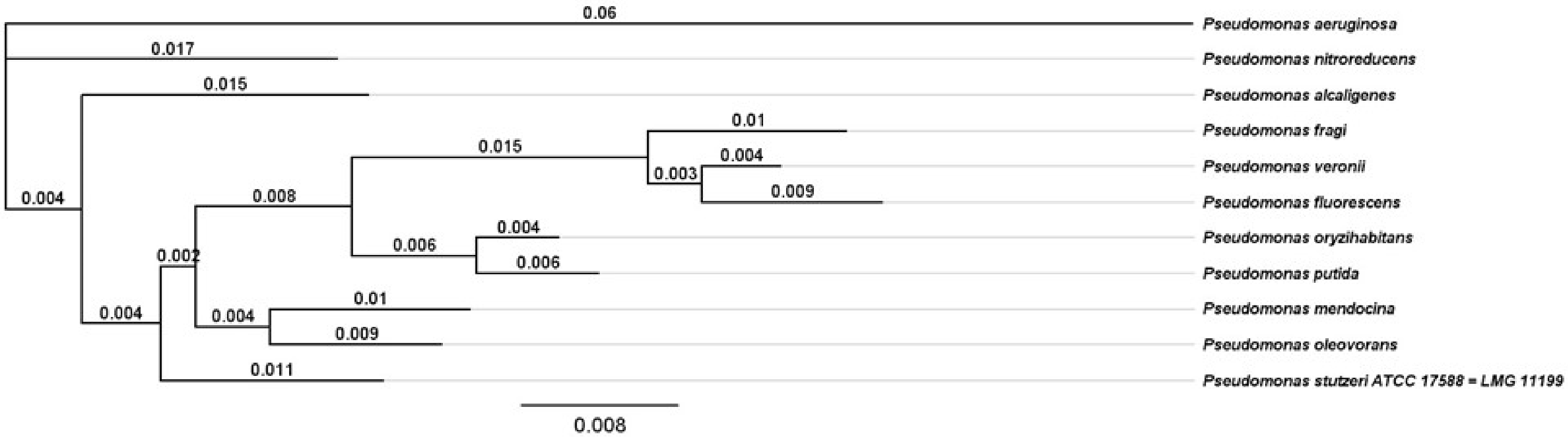

Supplementary Materials 7: Comparison of the phylogenetic relatedness of 10 non-*aeruginosa* species and *P. aeruginosa* from a Neighbor-Joining Tree of 16S rDNA sequence homology.
