# Supplementary figures and images for: The Microbiology of Non-aeruginosa Pseudomonas Isolated From Adults With Cystic Fibrosis: Criteria to Help Determine the Clinical Significance of Non-aeruginosa Pseudomonas in CF Lung Pathology
Source: Br J Biomed Sci. 2022 Jun 8;79:10468. doi: 10.3389/bjbs.2022.10468 (PMC9302546; doi:10.3389/bjbs.2022.10468)

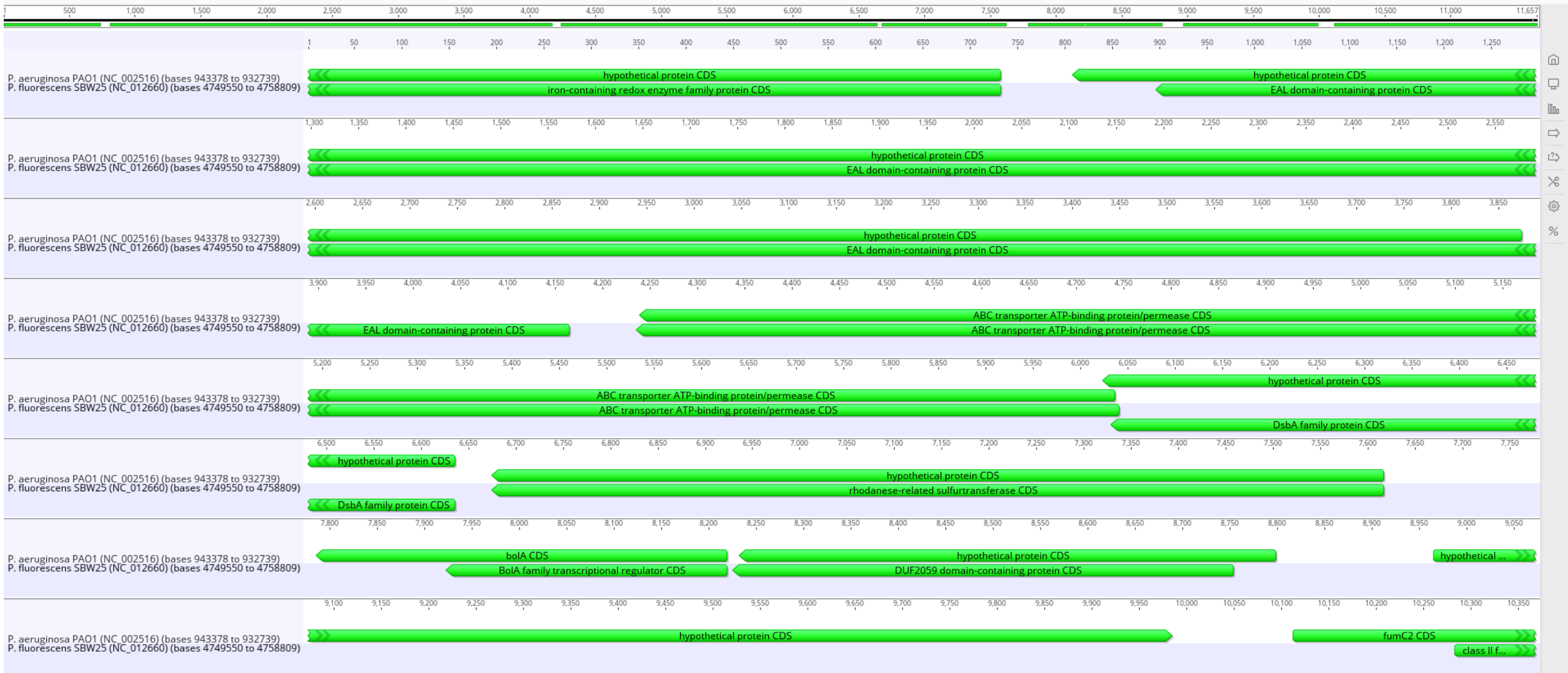

**Supplementary Materials 13:** Alignment of annotations in locally collinear block #27.

Supplement: Supplementary file 2 [file datasheet13.pdf]

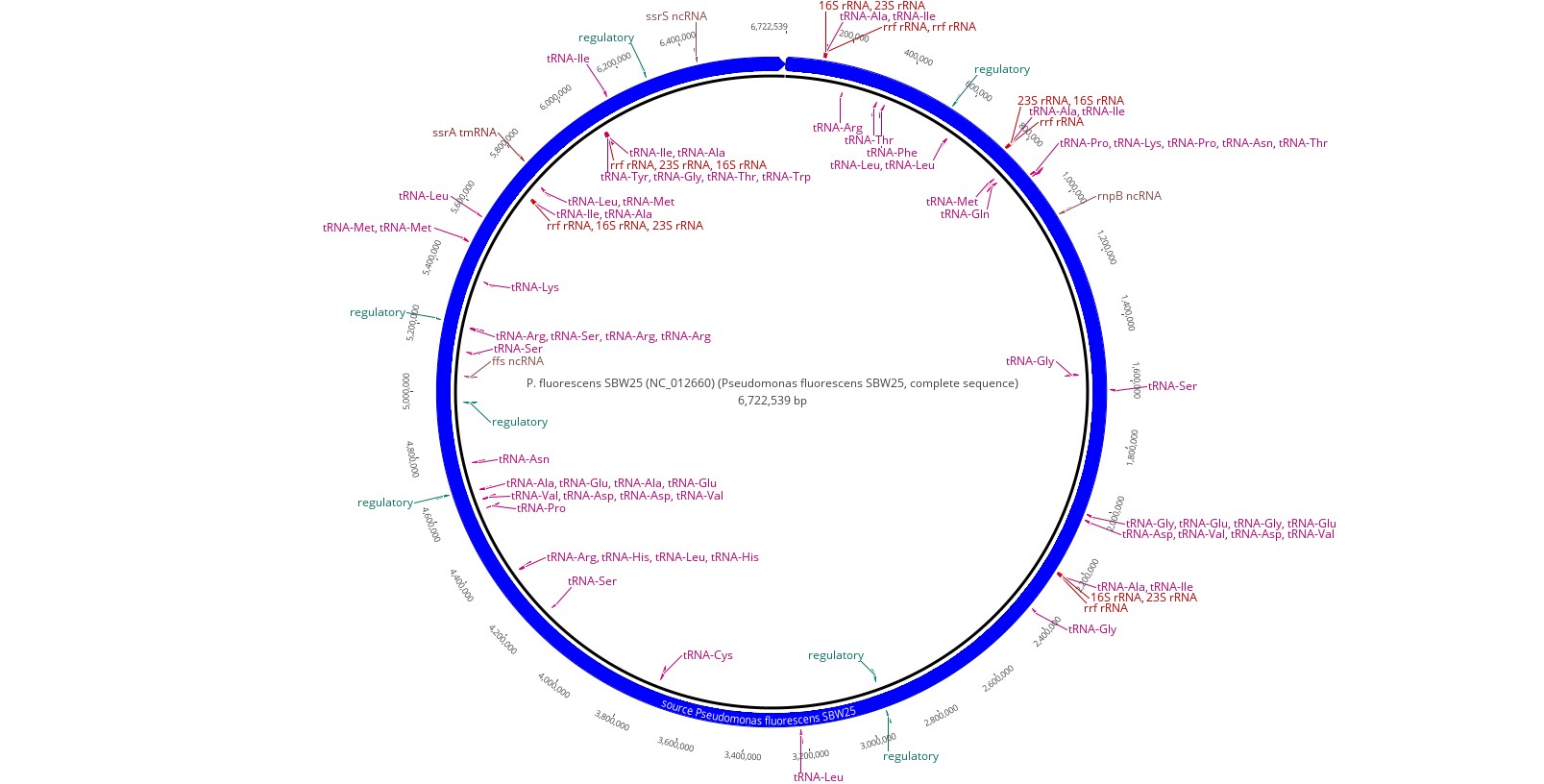

Supplement: Supplementary file 7 [file image9.jpeg]

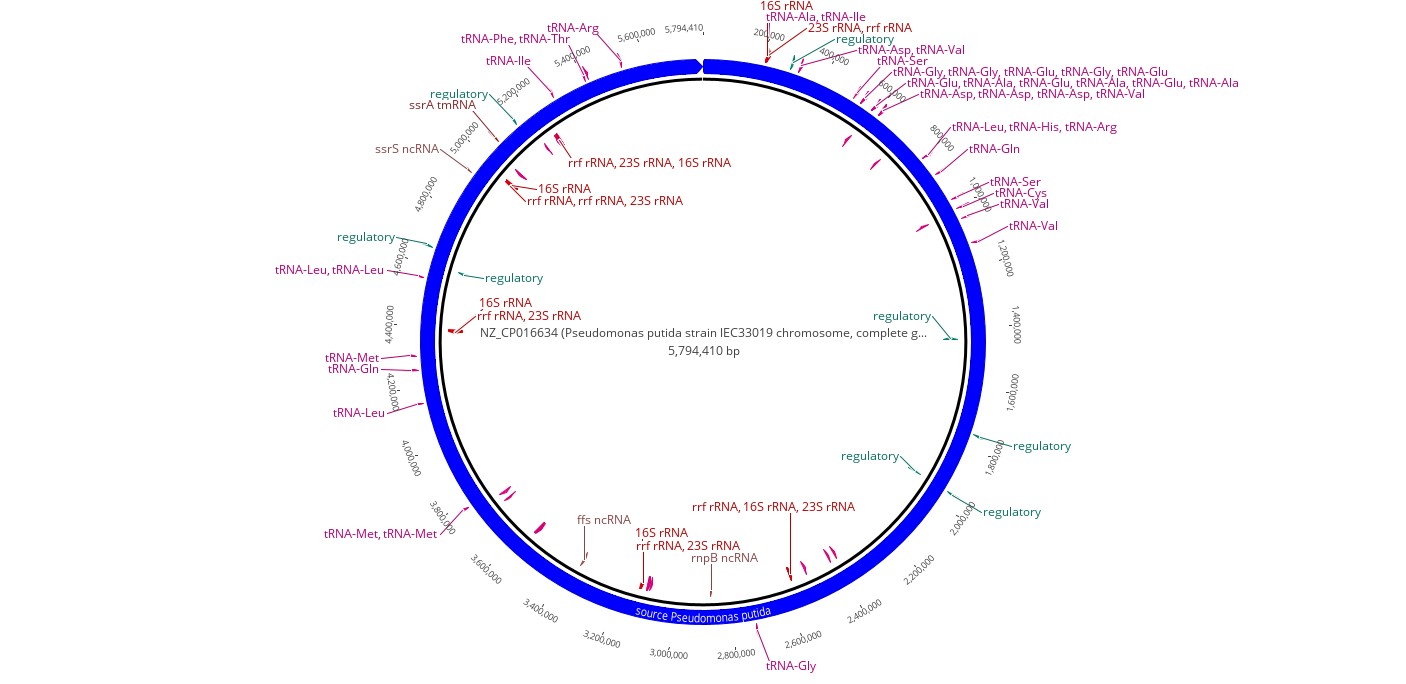

Supplement: Supplementary file 8 [file image10.jpeg]

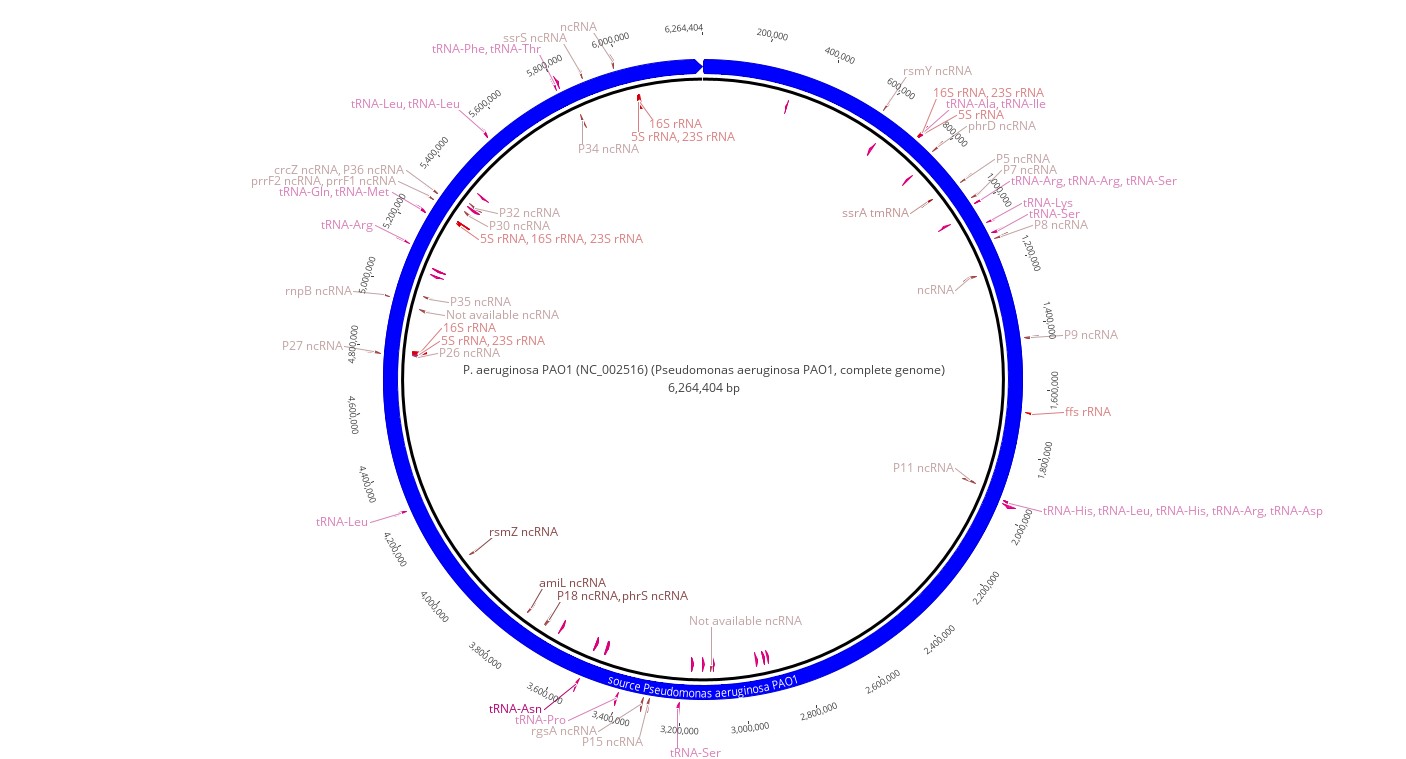

Supplement: Supplementary file 9 [file image12.jpeg]

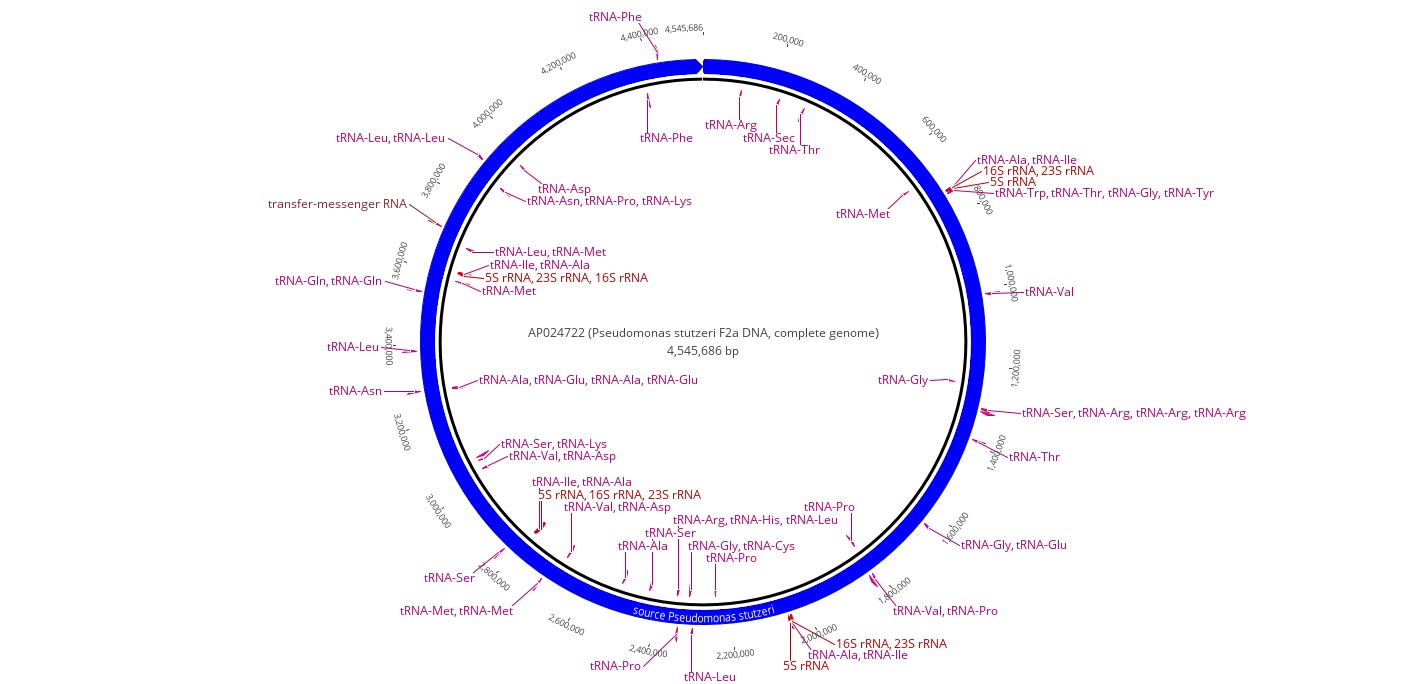

Supplement: Supplementary file 10 [file image11.jpeg]
