# Supplementary material for: The Microbiology of Non-aeruginosa Pseudomonas Isolated From Adults With Cystic Fibrosis: Criteria to Help Determine the Clinical Significance of Non-aeruginosa Pseudomonas in CF Lung Pathology
Source: Br J Biomed Sci. 2022 Jun 8;79:10468. doi: 10.3389/bjbs.2022.10468 (PMC9302546; doi:10.3389/bjbs.2022.10468)
Supplement: Supplementary file 3 [file datasheet2.pdf]

Superkingdom	Bacteria;
Phylum	*Proteobacteria;*
Class	*Gammaproteobacteria;*
Order	*Pseudomonadales;*
Family	*Pseudomonadaceae;*
Genera	*Pseudomonas;*
Species	*Pseudomonas alcaligenes*
	Pseudomonas fluorescens
	Pseudomonas fragi
	Pseudomonas mendocina
	Pseudomonas nitroreducens
	Pseudomonas oleovorans
	Pseudomonas oryzihabitans
	Pseudomonas putida
	Pseudomonas stutzeri
	Pseudomonas veronii

Supplementary Materials 2: Taxonomic description/lineage of the 10 *Pseudomonas* species isolated from adult patients with cystic fibrosis (CF)
