# Supplementary material for: The Microbiology of Non-aeruginosa Pseudomonas Isolated From Adults With Cystic Fibrosis: Criteria to Help Determine the Clinical Significance of Non-aeruginosa Pseudomonas in CF Lung Pathology
Source: Br J Biomed Sci. 2022 Jun 8;79:10468. doi: 10.3389/bjbs.2022.10468 (PMC9302546; doi:10.3389/bjbs.2022.10468)
Supplement: Supplementary file 5 [file datasheet6.pdf]

Supplementary Materials 6: Comparison of cultural, genotypic and pathological characteristics of the 10 non-*aeruginosa Pseudomonas* (NAP) isolated from the sputum of adulys with cystic fibrosis (CF).

**Characteristic**	***P. fluorescens***	***P. putida***	***P. stutzeri***	***P. alcaligenes***	***P. fragi***	***P. mendocina***	***P. nitroreducens***	***P. oleovorans***	***P. oryzihabitans***	***P. veronii***
**Description**	flu.o.res'cens. L. *Fluor* a flux.adj. *fluorescens* fluorescing	pu'ti.da L. adj. *putidus* stinking, fetid	stut'ze.ri Named after Dr A Stutzer, who named the species, M.L. gen. noun *stutzeri* of Stutzer	al.ca.li.ge.nes. French masc. n. *alcali*, alkali; from Arabic article *al*, the; from Arabic masc. n. *qaly*, ashes of saltwort; Gr. suff. *-genes*, producing; from Gr. ind. v. *gennaô*, to produce; N.L. part. adj. *alcaligenes*, alkali-producing	fra'gi. L. neut.n.*fragum* strawberry; L. gen.n. *fragi* of the strawberry	N.L. fem. adj. *mendocina*, pertaining to Mendoza (Argentina)	ni.tro.re.du'cens. Gr. neut. n. nitron, nitre, nitrate; L. pres. part. reducens, drawing backwards, bringing back to a state or condition; N.L. part. adj. *nitroreducens*, nitrate reducing	o.le.o'vor.ans L. *oleum* oil; L.v. *voro* to destroy, consume: M.L. part.adj. *oleovorans* oil-consuming	o.ry.zi'ha.bi.tans. L. fem. n. *oryza*, rice; L. pres. part. *habitans*, inhabiting; N.L. part. adj. *oryzihabitans*, rice inhabiting	masc. gen. n. *veronii*, of Veron, in honor of Prof. M. M. Veron, an eminent French microbiologist, for his contribution to taxonomy and medical microbiology
**Morphology**	Rods, 0.3-0.5 by 1.0 - 1.8 μm, occurring singly or in pairs. Motile, possessing a polar flagellum; occasionally non-motile	Rods with rounded ends. Motile, possessing polar flagella	Rods, 0.5 to 0.8 by 1.0 to 3.0 μm. Motile, possessing a single polar flagellum. Strongly coherent to agar media, becoming mucoid.	Gram-negative, straight and curved rods; motile by means of polar flagella; no indol production; milk alkaline; no fermentation of any carbohydrates.	Rods, 0.5 to 1.0 by 0.75 to 4.0 μm, occurring singly, in pairs and in chains. Motile with a polar flagellum	0.75 to 0.85 μm. wide and 1-4 to 2-8 μm. long in exponentially growing cultures. with a single polar or subpolar flagellum and a wavelength of 1.75 to 2.0 μm.	Rods, 0.4 to 0.6 by 1.4 to 1.8 μm. Occurring singly, rarely in pairs. Motile with polar flagella	Short rods, 0.5 to 0.8 to 1.5 μm, occurring singly and in pairs. Motile.	Rods 0.8 by 2.0 μm and have rounded ends. The cells occur singly, rarely in pairs and are motile by means of a polar monotrichous flagellum	Motile by means of a single polar flagellum
**Optimum temperature**	20 - 25ºC	25ºC Will grow at 37ºC	35ºC	25ºC	Grows from 10ºC to 30ºC. No growth at 37ºC. Very sensitive to heat.	37ºC. Can grow at 41ºC, but not at 4ºC.	25°C to 30°C. No growth at 37°C.	Good growth at 25ºC and 37ºC	30ºC	Growth occurs between 4ºC - 36°C but not at 41°C
**Nitrates reduced to nitrites**	Yes	Yes	Nitrates, nitrites, nitramines and N_2O reduced to elemental nitrogen	Yes	No	Yes	Yes	Yes	Yes	
**Habitat**	Soil, water, foodstuffs	Isolated from putrid materials, water	Isolated from soil. Found widely distributed in soil, manure, mud and stagnant water	Swimming pool waters	Isolated from milk and other dairy products, dairy utensils, water. Habitat is soil and water. Widely distributed.	Water and soil	Oil brine	Oil-soaked soils. Abundant in cutting compounds.	Rice paddy fields	Natural mineral water

**Pathological association with other infections**	bacteraemia, ocular infection,	bacteraemia, soft tissue infection, endocarditis, conjunctivitis, diabetic foot gangrene, catheter-associated bloodstream infection	prosthetic joint infection, bloodstream infection, intranasal infection, corneal infection, urinary tract infection	Wounds, endocarditis, bloodstream infection, fish pathogen	None	bacteraemia, endocarditis, sepsis, central nervous system infections and skin and soft tissue infections (SSTIs).	pneumonia	None	sepsis, peritonitis, endophthalmitis, and bacteremia.	Unknown
**% G+C**	60.1	61.6	62.4	65.8	58.6	62.8-64.3	65.2	64.5	65.1	61.5
**Genome size (Mb)**	6.3	6.2	4.5	4.4	5.1	5.4	6.8	5.57	5	6.9
**GenBank Accession numbers**	JRXT00000000, JRXU00000000, JRXV00000000, JRXW00000000, JRXX00000000, JRYA00000000, JRXY00000000	NZ_LNNI00000000.1	GCF_019704535.1	GCF_001597285.1	GCF_016812215.1	GCF_000725105.2	GCF_012986205.1	GCF_000732445.1	GCF_007665635.1	GCF_002028325.1
