# Supplementary material for: The Microbiology of Non-aeruginosa Pseudomonas Isolated From Adults With Cystic Fibrosis: Criteria to Help Determine the Clinical Significance of Non-aeruginosa Pseudomonas in CF Lung Pathology
Source: Br J Biomed Sci. 2022 Jun 8;79:10468. doi: 10.3389/bjbs.2022.10468 (PMC9302546; doi:10.3389/bjbs.2022.10468)
Supplement: Supplementary file 6 [file datasheet14.pdf]

Supplementary Materials 14: Annotations of coding DNA sequences (CDS) from Locally Collinear Block (LCB) #27 of a genome comparison between *Pseudomonas aeruginosa* PAO1 (NC_002516) and *Pseudomonas fluorescens* (SBW25 (NC_012660)

**Sequence Name**	**Name**
P. aeruginosa PAO1 (NC_002516) (bases 943378 to 932739)	ABC transporter ATP-binding protein/permease CDS
P. fluorescens SBW25 (NC_012660) (bases 4749550 to 4758809)	ABC transporter ATP-binding protein/permease CDS
P. aeruginosa PAO1 (NC_002516) (bases 943378 to 932739)	bolA CDS
P. fluorescens SBW25 (NC_012660) (bases 4749550 to 4758809)	BolA family transcriptional regulator CDS
P. fluorescens SBW25 (NC_012660) (bases 4749550 to 4758809)	class II fumarate hydratase CDS
P. fluorescens SBW25 (NC_012660) (bases 4749550 to 4758809)	DsbA family protein CDS
P. fluorescens SBW25 (NC_012660) (bases 4749550 to 4758809)	DUF2059 domain-containing protein CDS
P. fluorescens SBW25 (NC_012660) (bases 4749550 to 4758809)	EAL domain-containing protein CDS
P. aeruginosa PAO1 (NC_002516) (bases 943378 to 932739)	fumC2 CDS
P. aeruginosa PAO1 (NC_002516) (bases 943378 to 932739)	hypothetical protein CDS
P. aeruginosa PAO1 (NC_002516) (bases 943378 to 932739)	hypothetical protein CDS
P. aeruginosa PAO1 (NC_002516) (bases 943378 to 932739)	hypothetical protein CDS
P. aeruginosa PAO1 (NC_002516) (bases 943378 to 932739)	hypothetical protein CDS
P. aeruginosa PAO1 (NC_002516) (bases 943378 to 932739)	hypothetical protein CDS
P. aeruginosa PAO1 (NC_002516) (bases 943378 to 932739)	hypothetical protein CDS
P. fluorescens SBW25 (NC_012660) (bases 4749550 to 4758809)	iron-containing redox enzyme family protein CDS
P. fluorescens SBW25 (NC_012660) (bases 4749550 to 4758809)	rhodanese-related sulfurtransferase CDS
