# Supplementary material for: The Microbiology of Non-aeruginosa Pseudomonas Isolated From Adults With Cystic Fibrosis: Criteria to Help Determine the Clinical Significance of Non-aeruginosa Pseudomonas in CF Lung Pathology
Source: Br J Biomed Sci. 2022 Jun 8;79:10468. doi: 10.3389/bjbs.2022.10468 (PMC9302546; doi:10.3389/bjbs.2022.10468)
Supplement: Supplementary file 12 [file datasheet1.pdf]

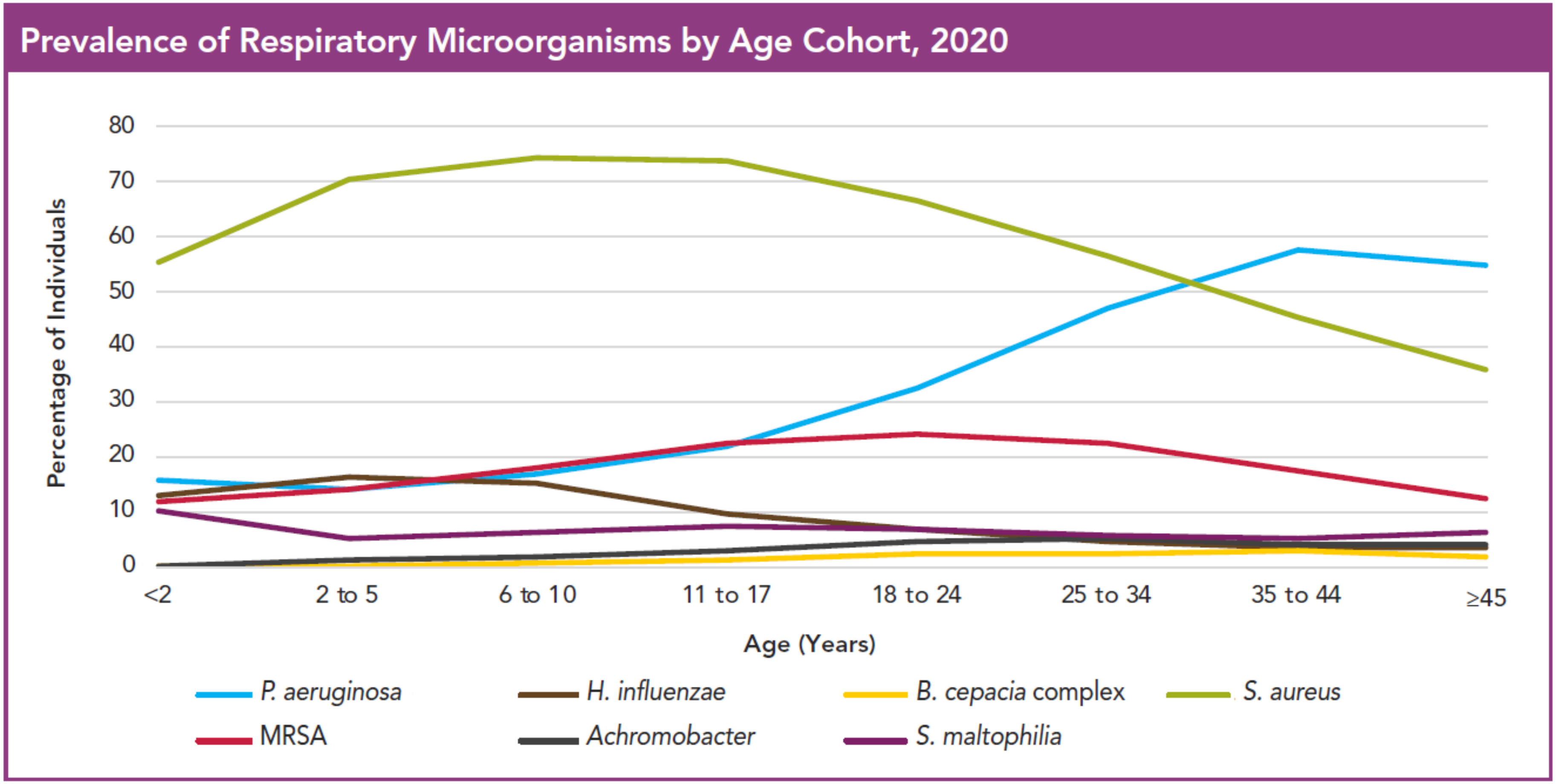

Supplementary Material #1: The most frequently isolated bacterial pathogens associated with respiratory infections in patients with cystic fibrosis. With permission. (SOURCE OF DATA: Cystic fibrosis patients under care at CF Foundation-accredited care centers in the United States, who consented to have their data entered). Cystic Fibrosis Foundation Patient Registry, 2020 Annual Data Report, Bethesda, Maryland, ©2021 Cystic Fibrosis Foundation).
