# Supplementary material for: The Microbiology of Non-aeruginosa Pseudomonas Isolated From Adults With Cystic Fibrosis: Criteria to Help Determine the Clinical Significance of Non-aeruginosa Pseudomonas in CF Lung Pathology
Source: Br J Biomed Sci. 2022 Jun 8;79:10468. doi: 10.3389/bjbs.2022.10468 (PMC9302546; doi:10.3389/bjbs.2022.10468)
Supplement: Supplementary file 13 [file datasheet5.pdf]

**Supplementary Materials 5:** **Combinations of two non-*aeruginosa Pseudomonas* species found in 14 Adult patients with cystic fibrosis.**

| Organism | *P. putida* | *P. stutzeri* | *P. alcaligenes* | *P. fragi* | *P. mendocina* | *P. oleovorans* | *P. oryzihabitans* |
|---|---|---|---|---|---|---|---|
| *Pseudomonas fluorescens* | 7 | 1 | 1 | 1 | 1 | | 1 |
| *Pseudomonas putida* | | 1 | | | | 1 | |
