# Supplementary material for: The Microbiology of Non-aeruginosa Pseudomonas Isolated From Adults With Cystic Fibrosis: Criteria to Help Determine the Clinical Significance of Non-aeruginosa Pseudomonas in CF Lung Pathology
Source: Br J Biomed Sci. 2022 Jun 8;79:10468. doi: 10.3389/bjbs.2022.10468 (PMC9302546; doi:10.3389/bjbs.2022.10468)
Supplement: Supplementary file 14 [file datasheet8.pdf]

Supplementary Materials 8: Alignment of the 16SrDNA gene from non-*aeruginosa* species of *Pseudomonas* isolated from adult people with cystic fibrosis (CF).

```
LOCUS       OM653502                1477 bp    DNA     linear   ENV 11-FEB-2022
DEFINITION  Uncultured Pseudomonas sp. clone CF 16S ribosomal RNA gene, partial
            sequence.
ACCESSION   OM653502
VERSION     OM653502
KEYWORDS    ENV.
SOURCE      uncultured Pseudomonas sp.
  ORGANISM  uncultured Pseudomonas sp.
            Bacteria; Proteobacteria; Gammaproteobacteria; Pseudomonadales;
            Pseudomonadaceae; Pseudomonas; environmental samples.
REFERENCE   1  (bases 1 to 1477)
  AUTHORS   Moore,J.E. and Millar,B.C.
  TITLE     Hypothetical 16S rDNA alignment of 11 species of Pseudomonas
            cultured from sputum of adult cystic fibrosis (CF) patients
  JOURNAL   Unpublished
REFERENCE   2  (bases 1 to 1477)
  AUTHORS   Moore,J.E. and Millar,B.C.
  TITLE     Direct Submission
  JOURNAL   Submitted (11-FEB-2022) Northern Ireland Public Health Laboratory,
            Belfast City Hospital, Lisburn Road, Belfast BT9 7AD, United
            Kingdom
COMMENT     ##Assembly-Data-START##
            Assembly Method       :: Geneious v. 2022-01-26
            Sequencing Technology :: gene alignment
            ##Assembly-Data-END##
FEATURES             Location/Qualifiers
     source          1..1477
                     /organism="uncultured Pseudomonas sp."
                     /mol_type="genomic DNA"
                     /isolation_source="sputum"
                     /host="Homo sapiens"
                     /db_xref="taxon:114707"
                     /clone="CF"
                     /environmental_sample
                     /country="United Kingdom"
     rRNA            <1..>1477
                     /product="16S ribosomal RNA"
ORIGIN
        1 gaactgaaga gtttgatcct ggctcagatt gaacgctggc ggcaggccta acacatgcaa
       61 gtcgagcgga tgagaggagc ttgctcctcg attcagcggc ggacgggtga gtaatgccta
      121 ggaatctgcc tggtagtggg ggataacgtt ccgaaaggaa cgctaatacc gcatacgtcc
      181 tacgggagaa agcaggggac cttcgggcct tgcgctatca gatgagccta ggtcggatta
      241 gctagttggt gaggtaatgg ctcaccaagg cgacgatccg taactggtct gagaggatga
      301 tcagtcacac tggaactgag acacggtcca gactcctacg ggaggcagca gtggggaata
      361 ttggacaatg ggcgaaagcc tgatccagcc atgccgcgtg tgtgaagaag gtcttcggat
      421 tgtaaagcac tttaagttgg gaggaagggc agtaagctaa taccttgctg ttttgacgtt
      481 accgacagaa taagcaccgg ctaactctgt gccagcagcc gcggtaatac agagggtgca
      541 agcgttaatc ggaattactg ggcgtaaagc gcgcgtaggt ggttygttaa gttggatgtg
      601 aaatccccgg gctcaacctg ggaactgcat ccaaaactgg ckagctagag tacggtagag
      661 ggtggtggaa tttcctgtgt agcggtgaaa tgcgtagata taggaaggaa caccagtggc
      721 gaaggcgacc acctggactg atactgacac tgaggtgcga aagcgtgggg agcaaacagg
      781 attagatacc ctggtagtcc acgccgtaaa cgatgtcaac tagccgttgg gatccttgag
      841 atcttagtgg cgcagctaac gcattaagtt gaccgcctgg ggagtacggc cgcaaggtta
      901 aaactcaaat gaattgacgg gggcccgcac aagcggtgga gcatgtggtt taattcgaag
      961 caacgcgaag aaccttacca ggccttgaca tgcagagaac tttccagaga tggattggtg
     1021 ccttcgggaa ctctgacaca ggtgctgcat ggctgtcgtc agctcgtgtc gtgagatgtt
     1081 gggttaagtc ccgtaacgag cgcaaccctt gtccttagtt accagcacgt watggtgggc
     1141 actctaagga gactgccggt gacaaaccgg aggaaggtgg ggatgacgtc aagtcatcat
     1201 ggcccttacg gcctgggcta cacacgtgct acaatggtcg gtacaaaggg ttgccaagcc
     1261 gcgaggtgga gctaatccca taaaaccgat cgtagtccgg atcgcagtct gcaactcgac
     1321 tgcgtgaagt cggaatcgct agtaatcgtg aatcagaatg tcacggtgaa tacgttcccg
     1381 ggccttgtac acaccgcccg tcacaccatg ggagtgggtt gctccagaag tagctagtct
     1441 tagccgtagg ggaacctgcg gctggatcac ctcctta
//
```
